# Renal Sympathetic Nerve-Derived Signaling in Acute and Chronic Kidney Diseases

**DOI:** 10.3390/ijms21051647

**Published:** 2020-02-28

**Authors:** Mi Ra Noh, Hee-Seong Jang, Jinu Kim, Babu J. Padanilam

**Affiliations:** 1 Department of Cellular and Integrative Physiology, University of Nebraska Medical Center, Omaha, NE 68198-5850, USA; mira.noh@unmc.edu (M.R.N.); heeseong.jang@unmc.edu (H.-S.J.); jinu.kim@jejunu.ac.kr (J.K.); 2 Department of Anatomy, Jeju National University School of Medicine, Jeju 63243, Korea; 3 Interdisciplinary Graduate Program in Advanced Convergence Technology & Science, Jeju National University, Jeju 63243, Korea; 4 Department of Internal Medicine, Section of Nephrology, University of Nebraska Medical Center, Omaha, NE 68198-5850, USA

**Keywords:** acute kidney injury, chronic kidney disease, sympathetic nervous system, norepinephrine, adrenergic receptor

## Abstract

The kidney is innervated by afferent sensory and efferent sympathetic nerve fibers. Norepinephrine (NE) is the primary neurotransmitter for post-ganglionic sympathetic adrenergic nerves, and its signaling, regulated through adrenergic receptors (AR), modulates renal function and pathophysiology under disease conditions. Renal sympathetic overactivity and increased NE level are commonly seen in chronic kidney disease (CKD) and are critical factors in the progression of renal disease. Blockade of sympathetic nerve-derived signaling by renal denervation or AR blockade in clinical and experimental studies demonstrates that renal nerves and its downstream signaling contribute to progression of acute kidney injury (AKI) to CKD and fibrogenesis. This review summarizes our current knowledge of the role of renal sympathetic nerve and adrenergic receptors in AKI, AKI to CKD transition and CKDand provides new insights into the therapeutic potential of intervening in its signaling pathways.

## 1. Introduction

Acute kidney injury (AKI) is associated with increased mortality and risk of development of chronic kidney disease (CKD) in the long term [1,2]. CKD is characterized by the persistent loss of renal function that frequently occurs over time [1]. Approximately 8–16% of the world’s population have advanced CKD [3], which has added complications of poor quality of life, financial burden, and demand for renal replacement therapy. In addition to renal deficiency, patients with CKD may have comorbidities including diabetes, hypertension, heart diseases, and stroke [1,2]. Despite multiple etiologies, the underlying pathophysiological process of CKD progression is fibrogenesis. Prevention of AKI and its potential progression to CKD is a challenging clinical problem [4]. There is no cure for CKD and no effective treatment for CKD has been developed. Despite being a major unmet medical need, current efforts are restricted to the control of blood pressure (BP) and optimization of renin–angiotensin–aldosterone system (RAAS) blockade. These therapies, at best, may reduce proteinuria, a surrogate marker of renal disease, but they only partially reduce progression of CKD [5].

The kidneys are abundantly innervated by both renal afferent sensory and efferent sympathetic nerves and communicate with the central nervous system via the sensory afferent nerves [6,7]. Increased renal afferent activity directly influences sympathetic outflow to the kidneys via efferent nerves [6,7,8]. The efferent sympathetic nerves are crucial for production of norepinephrine (NE), a key effector of the sympathetic nervous system [9,10]. Epinephrine and NE act by binding to adrenergic receptors (ARs), classified as α_1_-, α_2_-, or β-AR [11]. The contribution of sympathetic nervous system to the progression of CKD, a condition commonly characterized by renal sympathetic nerve hyperactivity, is well known [12,13,14]. Increased plasma NE is observed in patients with CKD and is strongly linked with the development of kidney injury [15]. Furthermore, previous studies have found that the infusion of NE into the renal artery causes ischemic renal injury by constricting renal vasculature [16,17,18]. The renal sympathetic nervous system has drawn increasing interest over many years, after the introduction of renal sympathetic denervation into clinical medicine [19,20]. Despite the recognition of the renal sympathetic nerve system as the effector of renal dysfunction in CKD, its role in the progression and development of CKD has not been well defined.

Increased renal sympathetic nerve activity leads to increased renin secretion, increased renal tubular sodium reabsorption, and decreased renal blood flow [6,10,21]. Renal denervation (RDNx) is proposed as a therapeutic strategy used in treatment of resistant hypertension [8,21,22]. RDNx performed surgically or chemically reduces sympathetic nerve activity and has been shown to reduce BP, improve renal function, and attenuate the progression of CKD in experimental models and humans of hypertension and CKD [23,24,25,26,27,28,29,30]. We reported that RDNx prevented the development of tubulointerstitial fibrogenesis and inflammation after unilateral ureteral obstruction (UUO) and kidney ischemia/reperfusion injury (IRI) independent of BP changes [25,31]. In addition, RDNx did not worsen kidney function of patients and renal injured mice—as assessed by glomerular filtration rate (GFR), plasma creatinine, cystatin C, or urea levels, and tubular morphological damages—indicating that it is safe even when CKD is present [25,31,32,33]. Here, we review the progress in our understanding of the molecular mechanisms of NE and ARs signaling in AKI, AKI to CKD transition and CKD.

## 2. Adrenergic Receptors and Norepinephrine in the Kidney

Norepinephrine (NE) is an organic chemical in the catecholamine family that functions in the body as the main neurotransmitter of sympathetic nerve system [34]. NE released from the nerve terminals, together with co-transmitters, such as ATP and neuropeptide Y, regulate kidney function [35]. NE in the kidney is involved in a number of physiological processes, including control of renal blood flow, glomerular filtration rate, reabsorption of water, sodium, and other ions, and release of renin [6]. We recently found that renal nerve-derived NE signaling via α_2_-ARs, promotes renal inflammation and interstitial fibrosis [25,31].

ARs, which are G protein coupled receptors, are well known to participate in various physiological renal functions [36,37,38,39]. Localization of ARs has been confirmed using various approaches including in situ hybridization, immunohistochemistry, and competitive binding assays [40,41,42,43]. The presence of adrenergic receptors in nephron segments, including proximal convoluted tubules and cortical and medullary collecting ducts, has previously been demonstrated (Table 1) [40,42,43,44]. In the kidney, α_1_-AR is expressed in arterioles, whereas α_2_-AR is expressed predominantly in proximal tubules [45]. α_1_-AR and α_2_-AR are responsible for stimulation of renal vasoconstriction and Na^+^ reabsorption, respectively [45]. β-ARs can be divided into three subtypes: β_1_-AR, β_2_-AR, and β_3_-AR [11] and are expressed in most of the nephron segments, including proximal tubules and distal convoluted tubules [38,43]. The roles of β_1_- and β_2_-ARs are well known for regulating renal blood flow, glomerular filtration rate (GFR), sodium and water reabsorption, acid-base balance, and secretion of renin in the kidney [38]. β_3_-AR is important for regulating renal water and solute reabsorption [38,46]. The renal localization of ARs and their role for the regulation of renal function have been reviewed in detail, largely based on data obtained in experimental animal models [36,37,39,46].

## 3. Renal Sympathetic Nervous System in AKI and CKD

Acute kidney injury (AKI) is defined as an abrupt or rapid decline in renal filtration function and is the result of combined acting of multiple factors [47,48]. Previous studies suggested that clinical AKI may initiate the onset of progressive renal diseases [49,50,51,52]. AKI can cause end-stage kidney disease (ESKD) directly, and increase the risk of developing CKD. Maladaptive repair after AKI may exceed the capacity of compromised renal reserve, resulting in the functional manifestation of CKD over time [53].

Renal sympathetic nervous system and circulating catecholamines including NE are considered to be involved in the development of the progressive renal tissue injury accompanying AKI [54,55]. In addition, NE injected into renal artery causes ischemic AKI with obstruction of tubule lumens, changes in renal hemodynamics and histological damage in the kidney [16,56,57]. Recent studies reported that renal ischemia is an important primary event leading to increased sympathetic nerve activity [58,59]. Renal venous plasma NE and renal tyrosine hydroxylase expression, which is related to NE synthesis, have been shown to increase after unilateral renal artery occlusion and bilateral renal ischemia/reperfusion (I/R) [58,60,61]. In our previous study, post-ischemic renal NE content increased up to day 16 after unilateral I/R [31]. Other studies reported that plasma and renal NE concentrations increased up to day 3 after bilateral I/R and returned to control level at day 7 [62]. Cisplatin, a widely used chemotherapeutic anti-cancer agent, induces nephrotoxicity and AKI [63]. Cisplatin-induced renal injury causes severe vasoconstriction and tissue damage, which activate renal sympathetic nerves system as shown by the increase in renal venous NE level after cisplatin administration and disrupt normal baroreflex, a reflex mechanism to maintain blood pressure (BP), and control of renal sympathetic nerve activity [64,65,66,67]. Indeed, cisplatin-induced renal failure is accompanied by reduced renal blood flow associated with increased renal vascular resistance [65,68,69]. 

Renal sympathetic nervous system contributes to pathogenesis of not only ischemic AKI, but also of CKD and hypertensive disease [34,70,71]. In our previous studies, it has been demonstrated that renal nerve-derived NE signaling induces fibrogenesis and inflammation in two different CKD models, UUO and unilateral I/R [25,31]. Sympathetic activity is already elevated in early phases of chronic renal failure [12,71]. These reports clearly demonstrate that enhanced renal sympathetic nerve activity and its consequent effects on NE overflow from nerve endings could play important roles in the development of AKI and its progression to CKD. Sympathetic nervous hyperactivity occurs in the CKD and hypertension, and the level of sympathetic nervous activity is associated with the increase of BP [72]. Increased renal sympathetic nerve activity enhances renin production and release leading to increased angiotensin II (Ang II), the main effector of the renin–angiotensin system (RAS) in hypertensive patients [73,74,75]. NE release mediates vasoconstriction of the renal vasculature, as well as sodium and water reabsorption at renal tubular epithelial cells, and renin release from the juxtaglomerular apparatus, which in turn results in increase of Ang II [76]. Conversely, Ang II can enhance NE release and reduce uptake of NE by acting on adrenergic nerve terminals, resulting in increased sympatho-excitation in the heart, kidney, and vasculature [8,77,78]. It has been proposed that the RAS activates the sympathetic nervous system and angiotensin-converting enzyme inhibition and angiotensin receptor blockade are antiadrenergic [78]. Obesity increases the susceptibility to AKI and subsequent progression to CKD [79,80]. Blockade using doxazosin, an α_1_-AR antagonist, clonidine, an α_2A_-AR agonist, and atenolol, a β_1_-AR antagonist, lowered BP and heart rate in obese dog and human patients [81,82,83]. In addition, RDNx attenuated sodium retention and prevented BP increase in a dog model of high fat diet-induced obesity [84]. There is evidence that angiotensin type 1 receptor interacts with α_2A_-AR. Genetic inhibition of α_2A_-AR or pharmacological inhibition of α_2_-AR lowers Ang II-mediated NE release in kidneys with 5/6 nephrectomy [85].

## 4. Inactivation of NE-AR Signaling in AKI and CKD

Several studies demonstrated beneficial effects of sympathetic nerve system inhibition on renal function and morphology. Clonidine and moxonidine, α_2_-AR agonist, and propranolol, β-AR antagonist, are known to have renoprotective actions against post-ischemic AKI [86,87,88]. We previously demonstrated that inhibition of α_2_-ARs prevents interstitial fibrogenesis after IRI, as indicated by reduced TGF-β1 production, Smad3 phosphorylation, α-SMA expression, and collagen deposition [31]. Other reports have indicated that inhibition of either α_1_-AR or β-AR protects the kidney against 5/6 nephrectomy-induced injury, and a combinational inhibition of α_1_-AR and β-AR is more effective in preventing renal injury than the inhibition of α_1_-AR or β-AR alone [89]. In uninephrectomized rats before ischemia and mice subjected to unilateral ischemia, yohimbine, and atipamezole, both α_2_-AR blockers and JP-1302, an α_2C_-AR specific blocker, mitigate I/R-induced renal tubular damage [31,90]. In the cisplatin-induced nephrotoxicity model, it has been reported that α_2_-AR inhibition with yohimbine and JP-1302 improves renal blood flow, suppresses increased plasma NE level, and ameliorates cisplatin-induced renal dysfunction and histological damages, by suppressing pro-inflammatory cytokine expression such as TNF- α and MCP-1 [91]. RDNx also restores baroreflex sensitivity to normal values, heart rate and BP in cisplatin-induce renal failure [64,92].

In addition, ischemic acute renal failure is ameliorated by RDNx or ganglionic blockade, which blocks the sympathetic nerves, and that effect is accompanied by suppression of elevated renal venous NE level immediately after reperfusion and attenuation of decreased GFR after the I/R [93]. Most studies reported that RDNx provides beneficial effects on BP of patients with stage 3–4 chronic kidney disease [94]. Accumulating evidence suggests that RDNx may help to delay the decline of renal function in chronic kidney disease [95,96]. Pharmacological denervation by DSP-4 also inhibits I/R-induced renal functional and morphological impairments [60]. Renalase is a monoamine oxidase secreted by the renal proximal tubules and degrades catecholamines including NE [97]. Prevented increase in plasma NE levels associated with I/R-induced renal cell death and kidney function impairment [98]. Genetic blockade of renalase increases plasma NE levels and suffers increased renal tubular injury with increased tumor necrosis factor-α (TNF-α), monocyte chemoattractant protein-1 (MCP-1), and MIP-2 after renal I/R. Administration of recombinant renalase suppresses NE overproduction and attenuates ischemic renal damage [98]. 

Experimental studies demonstrated that renal denervation slowed or delayed the rise in BP in experimental models of hypertension such as spontaneous hypertension, salt-sensitive hypertension, Nω-nitro-L-arginine methyl ester (L-NAME) induced hypertension, Ang II induced hypertension and deoxycorticosterone acetate (DOCA)-salt hypertension [99,100,101,102,103,104,105]. Singh et al. reported that the reduction in BP and albuminuria and improved GFR in sheep with hypertensive CKD, observed at 5 months after catheter-based RDNx, were sustained until 30 months after RDNx, demonstrating the long-term durability of the beneficial effects of RDNx in hypertensive CKD [106]. Although the effect of RDNx is associated with suppressed RAS components expression, it remains to be defined whether renal sympathetic nerves or related signaling control RAS in CKD and hypertension progression through NE and adrenergic receptor. 

On the other hand, activation of AR using agonist has protective effect in the kidney. It has been confirmed that dexmedetomidine, a selective α_2_-AR agonist, exerts notable positive effects in inhibiting sympathetic nerves and plasma NE levels and preventing kidney damage [107,108,109,110]. Dexmedetomidine effectively inhibits sympathetic activity and plays an important role in kidney protection by reducing inflammation, oxidative stress, and apoptosis through activating α_2_-AR and down-regulating TLR4 expression in I/R-induced [110] and LPS-treated mice [108]. Dexmedetomidine down-regulates the apoptosis of kidney tubular epithelial cells by inhibiting activation of signaling pathways, JAK/STAT and MAPK in I/R-induced rats [109]. Noh et al. have demonstrated that β_2_AR agonists have an anti-inflammatory action that targets macrophage activation in diabetic rats, especially in the kidney [111]. Overexpression of β_2_-AR using gene transfer with human β_2_-AR was effective in preventing endotoxin-induced renal injury with reduced elevation of TNF-α mRNA and leukocyte infiltration into the rat kidney [112].

## 5. Mechanisms of NE-AR Signaling in AKI and CKD

The mechanisms underlying AKI to CKD progression involves multiple interactions between injured tubules, immune cells, endothelial cells, and fibroblasts [113]. The importance of inflammation in the development and progression of kidney fibrosis is well established [113]. Once the kidney is injured, inflammatory cells infiltrate to the injured site and subsequently precede kidney fibrosis through production and release of profibrotic cytokines and growth factors [114]. These profibrotic cytokines and growth factors contribute to recruitment and activation of myofibroblasts, which cause progressive interstitial fibrosis, leading to CKD [114,115]. Renal efferent sympathetic nerves reportedly mediate renal inflammation through the trafficking and activation renal inflammatory immune cells and releasing inflammatory chemokine and cytokine content [116]. Le Clef et al. reported that the expression level of inflammatory cytokines, TNF-α and interleukin-6 (IL-6), remained higher with longer ischemia times [117]. The activation of α_2_-AR enhances the progression of dysfunction and inflammatory responses in the kidney [118]. Activation of α_2_-AR is responsible for upregulation of inflammatory cytokines including TNF-α and IL-6 and enhancement of granulocyte and monocyte recruitment [118]. NE or the α_2_-AR agonist UK-14304, significantly elevates LPS-induced TNF production from macrophages via α_2_-AR located on macrophages, while β-adrenergic agonist, isoproterenol, inhibits TNF release from macrophages [119,120,121]. In addition, inhibition of α_2C_-AR with α_2c_-AR antagonist JP-1302, after I/R, strongly suppressed cytokines, TNF-α, IL-6, IL-1β, and MCP-1 expression [122]. These increased inflammatory cytokines, IL-1β, IL-18, IL-6, and TNF-α recover at week 2 after RDNx [20]. During kidney interstitial fibrogenesis after UUO and IRI, RDNx attenuates the infiltration of neutrophils and macrophages [25,31]. In addition, it has been reported that RDNx reduces the accumulation of leukocytes, T cells, and both CD4+ and CD8+ T cells in the kidney of Ang II-treated hypertensive mice [116], decreases the activation of monocyte, monocyte-platelet aggregates formation, and plasma levels of monocyte-related cytokines and chemokines, MCP-1, IL-1 β, TNF- α, and IL-12 in human hypertensive patients [123]. NE has been implicated in promoting inflammatory cytokines release and production and myeloid cell recruitment into the injured site [124,125]. We previously reported that increased NE concentration correlated with the levels of inflammatory cytokines and with cell damage, in UUO or the I/R-induced mice model [25,31]. Furthermore, administration of α_2_-AR antagonists has no effect on NE level but decreases cytokine and chemokine expression after UUO, suggesting that norepinephrine may promote leukocyte recruitment and inflammation [25,31]. Together, these results suggest that following an injury stimulus, NE-AR signaling drives the inflammatory response, which in turn contributes to the development or progression of CKD.

Another potential mechanism for sympathetic nerve system activation in the AKI and CKD model is reduced nitric oxide (NO) availability [77]. In the kidney, NO has numerous physiological roles including the modulation of renal sympathetic nerve activity [126,127]. Ischemic AKI has been shown to alter renal hemodynamics, linked to endothelial cell dysfunction caused by increased reactive oxygen species (ROS) production, leading to decreased NO availability [128]. In addition, renal NO synthase activity is progressively reduced in the chronic renal failure model due to decreasing NO formation [129]. Accumulation of L-arginine analogues, NO synthase inhibitors, in CKD could potentially directly increase sympathetic nervous system (SNS) activity by inhibiting NO production [14]. Inhibition of NO synthesis induces vasoconstriction of the glomerular microvasculature and causes proximal tubular reabsorption to decline [14]. These effects are prevented by prior RDNx or the administration of an α_2_-ARantagonist [130,131]. Administration of an α_2_-AR agonist, B-TH933 to the denervated kidney restores both glomerular and tubular responsiveness to NO inhibition [44]. Decreased NOS activity induced by L-NAME, one of the non-selective NO synthase inhibitors, was significantly restored by α_1_-AR blockers, prazosin, or doxazosin, and attenuation of L-NAME-induced renal injury [132,133,134]. β-blockers, such as carvedilol and nebivolol, are shown to have protective effects via stimulation of NO release with protecting the kidney from 5/6 nephrectomy, gentamycin-induced nephrotoxicity, or hypertensive model [135,136,137]. 

Kidney tubular cell cycle arrest induced by severe AKI contributes to renal fibrogenesis by releasing profibrotic cytokines such as TGF-β1 and connective tissue growth factor (CTGF) [138]. While cell cycle arrest is normally used as a protective mechanism to avoid cell division during stress and injury, sustained cell cycle arrest at the G2/M phase results in the activation of senescence-associated secretory phenotype (SASP) and c-jun NH2-terminal kinase (JNK) signaling cascade, leading to the synthesis and the secretion of proliferative and profibrotic factors, which induce fibroblast proliferation and collagen deposition in renal interstitium [138,139]. In a previous study, it has been reported that RDNx prevents tubular cell cycle arrest at the G2/M phase observed during fibrogenesis after IRI with decreased number of tubular epithelial cells positive for phosphorylated histone H3, markers of the G2/M and decreased ratio of cyclin B1 to cyclin D1, markers of the G2/M cell cycle arrest [31]. However, in the denervated kidney, administration of NE induces cell cycle arrest at the G2/M phase, activates TGF- β1 signaling and increases α-SMA expression [31]. These suggest that renal nerve-derived NE may contribute to cell cycle arrest, leading to renal fibrogenesis. 

The pathophysiology of AKI is generally characterized by a common cascade of cell death-induced inflammation and fibrosis following injury [140]. Inhibition of cell death attenuates renal inflammation and the consequent renal fibrogenesis after kidney injury [141,142,143,144]. Our recent study investigating the role of renal sympathetic nerves in renal fibrogenesis after renal I/R demonstrated that unilateral RDNx at the time of injury, or up to 1-day post-injury, improved histology, decreased pro-inflammatory and pro-fibrotic responses and apoptosis, thereby indicating that renal nerve stimulation is a primary mechanism and renal nerve-derived factors drive epithelial cell cycle arrest and the inflammatory cascade causing interstitial fibrogenesis after ischemia reperfusion injury [31]. NE can induce apoptosis by activating the ROS/JNK signaling pathway [145]. In cultured kidney proximal tubule cells, NE treatment increases poly (ADP-ribose) polymerase 1 (PARP1) and cleaved caspase-3 expression, which is reduced by co-treatment with caspase-3 inhibitor [59]. Consistent with our data, RDNx inhibits renal sympathetic activation, significantly attenuating the expression levels of p53, TNF-α, NF-κB, caspase-2 and -3, and improving apoptosis [146]. On the other hand, activation of α_2_-AR with dexmedetomidine, α_2_-AR agonist attenuates renal cell apoptosis and renal tubule impairment by inhibiting mitochondrial apoptosis pathways in the sepsis- or I/R-induced injury model, which may be effective by regulating NE release [147,148,149]. These findings indicate that inhibiting NE-AR signaling may attenuate apoptosis and may represent one of the mechanisms by which NE-AR signaling may prevent or limit progression of renal fibrogenesis at its onset in AKI-induced CKD.

## 6. Role of Sympathetic Nerves in Other Organs

Heart failure is a disorder in which the heart is unable to pump an adequate supply of blood [150]. SNS overactivity is associated with an increased risk of cardiovascular mortality in patients with renal failure (Table 2) [151]. Sympatho-excitation is a major component of the pathological relation between the kidney and heart in congestive heart failure [150]. It has been reported that the efferent renal nerves are essential to the renal hypoperfusion in congestive heart failure [152]. Furthermore, NE is released from the heart with an impaired NE re-uptake [153,154], thereby producing direct effects on β-AR signal transduction and impaired inotropic stimulation of the heart [155]. RDNx showed improvement of sodium excretion in experimental heart failure [156], increased cardiac output, improved renal blood flow [157], and a down-regulation of angiotensin II type 1 receptors mediating maladaptive responses [158]. Linz et al. have suggested that RDNx attenuates malignant ventricular arrhythmias in pigs with ventricular ischemia [159]. Similarly, RDNx improved ventricular end-diastolic diameter and fractional shortening in rats with heart failure induced by myocardial infarction [160]. It has been reported that α_2_-ARs play essential roles in the prevention of heart failure [161]. α2-AR subtypes are the presynaptic inhibitory receptor controlling sympathetic NE release [10]. Deletion of α_2A_-AR and α_2C_-AR subtypes increased the susceptibility to develop heart failure following chronic pressure overload in vivo [161,162]. In accordance with this, activation of α_2A_-AR using α_2A_-AR agonists, clonidine and moxonidine though sympatho-inhibition effect protect the kidney against renal IRI [87,163]. Conversely, in our previous reports, we demonstrated that the activation of the α_2_-AR accelerates the progression of renal fibrogenesis after renal IRI and UUO, suggesting the activation of α_2_-AR in this model may trigger inflammatory and fibrotic signaling pathways than sympatho-inhibition to induce fibrogenesis [25,31]. The contrasting results in the heart failure models versus the renal and IRI and UUO models could be due to the fact that in the mouse heart failure model, deletion of sympatho-inhibitory α_2_-ARs results in increased norepinephrine levels, which elicit typical cardiovascular consequences, including aggressive remodeling of the left ventricle, cardiac hypertrophy, and fibrosis [161,164]. In the renal denervated kidney, administration of exogenous NE can promote leukocyte recruitment, activate the TGF-β1 signaling pathway, and induce G2/M cell cycle arrest [25,31].

Hepatorenal syndrome (HRS) is currently defined as the occurrence of AKI, in patients with advanced chronic liver disease [165,166]. Since catecholamines are metabolized by the liver, elevated arterial plasma NE level in cirrhosis might be due to impaired hepatic function (Table 2). With increasing severity of cirrhosis, the sympathetic nerves are progressively activated with increased circulating and renal NE concentrations [167,168,169]. Enhanced sympathetic nervous activity and NE is important in maintaining the level of arterial blood pressure in cirrhosis [170]. The treatment of HRS using α_1_-AR agonist, midodrine administration is associated with a significant improvement in renal function in HRS patients [171]. Other α_2_-AR agonist, clonidine, reduces portal pressure, arterial BP, and circulating NE in patients with cirrhosis [172]. On the other hand, in end-stage cirrhosis, β-blockers may reduce survival due to their negative effect on the cardiac compensatory reserve [173,174]. β-blockers are associated with increased mortality in patients with refractory ascites at high risk of paracentesis-induced circulatory dysfunction [173]. For this reason, these studies suggest that β-blockers should be avoided in patients with both cirrhosis and renal failure. Moreover, renal vasoconstriction caused by RAS activation and sympathetic nervous systems is the primary cause of HRS [175]. As mentioned above, Renin, a major RAS component, is produced in the juxtaglomerular cells of the afferent renal arteriole, reduced sodium intake, and increased activity of the sympathetic nervous system [78]. RAS activation is one of the main factors associated with renal vasoconstriction in HRS [176,177]. Thus, sympathetic nervous overactivity plays an important role in chronic liver disease.

## 7. Conclusions

**Figure 1:**
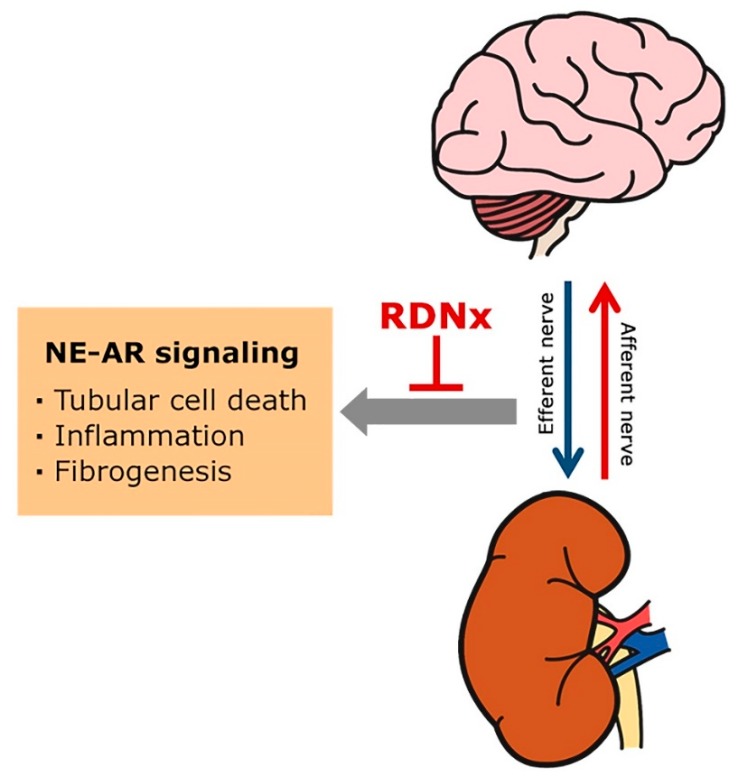
Renal sympathetic nerve-derived norepinephrine (NE) and adrenergic receptor (AR) in chronic kidney disease development and progression. Sympathetic efferent activation, which is generated in the central nervous system, contributes to sympatho-excitation in the vasculature and renal tubules, and mediate their effects via norepinephrine and adrenergic receptors (ARs). The increased renal sympathetic nerve-derived NE may trigger tubular cell death, renal inflammation, and fibrogenesis progression, leading to CKD. Renal denervation (RDNx) lowers NE in renal tissue after renal injury and has a protective effect against fibrogenesis and CKD progression.

The role of the sympathetic nervous system in AKI and CKD are discussed through a literature review investigating sympathetic nervous mechanisms [14,39,58,96]. As discussed, various studies have proposed a role for the renal sympathetic nervous system in contributing to renal interstitial inflammation and fibrogenesis in AKI and CKD. The renal sympathetic nerve-derived NE mediates inflammatory and fibrogenic response, and blockade of NE-AR signaling can prevent renal interstitial fibrogenesis following kidney injury (Figure 1). In addition, accumulating evidence suggested that renal denervation can help to delay reduction of renal function in CKD [31,178,179]. However, the NE-AR signaling pathway leading to renal injury in AKI and CKD remains to be defined. Thus, further research is needed to investigate the mechanisms by which renal sympathetic nerve-derived signaling may lead to progressive CKD. Defining the key molecules and understanding their functions may lead to designing of novel therapeutic strategies to prevent the progression of AKI to CKD.

## Figures and Tables

**Table 1:** Localization and action of adrenergic receptors in the kidney.

Subtype	Localization in Kidney	Action
α_1_-AR	Arterioles	▪ Stimulation of renal vasoconstriction
α_2_-AR	Proximal tubules	▪ Stimulation of Na^+^ reabsorption
β-AR	All nephron segments	▪ Regulation of renal blood flow ▪ Regulation of glomerular filtration rate ▪ Reabsorption of Na^+^ and water ▪ Secretion of renin

**Table 2:** Activated sympathetic nerve in other organs.

Organ	Consequences
Heart	▪ Increased cardiovascular mortality ▪ Impaired NE re-uptake ▪ Impaired inotropic stimulation
Liver	▪ Increased arterial blood pressure ▪ Activated Renin-Angiotensin system ▪ Increased renal vasoconstriction
